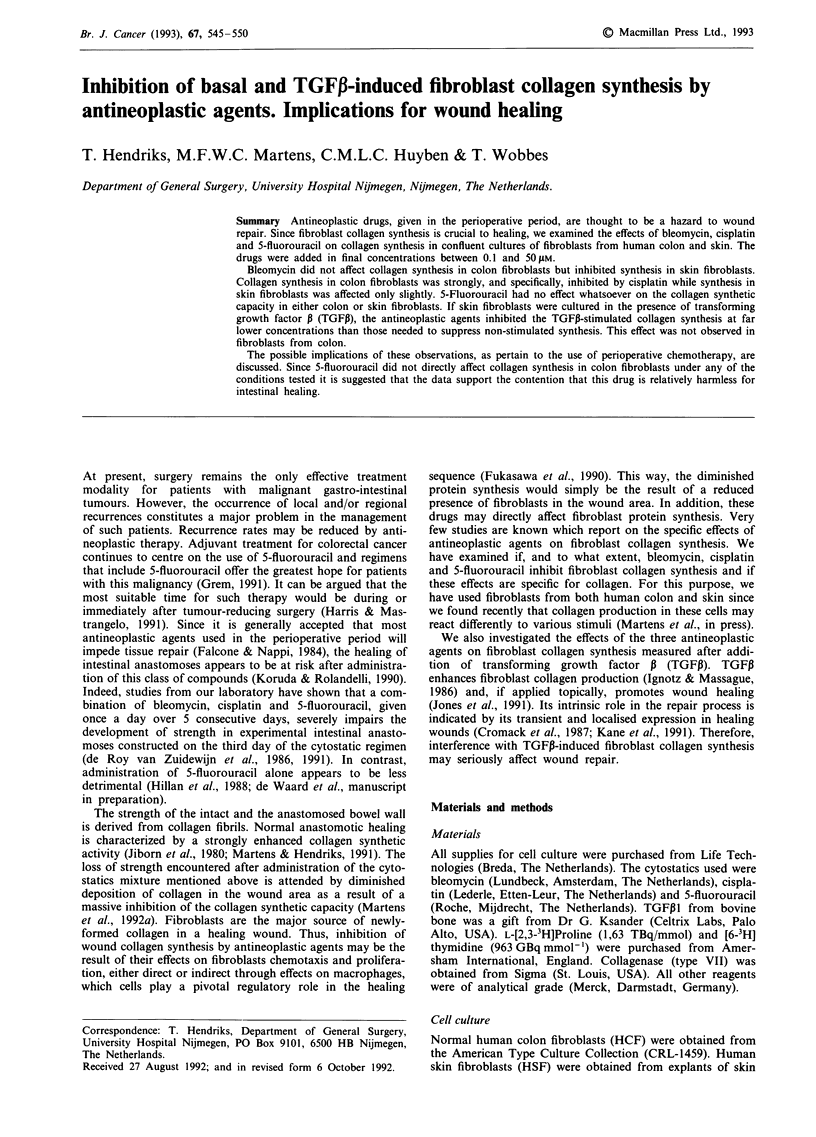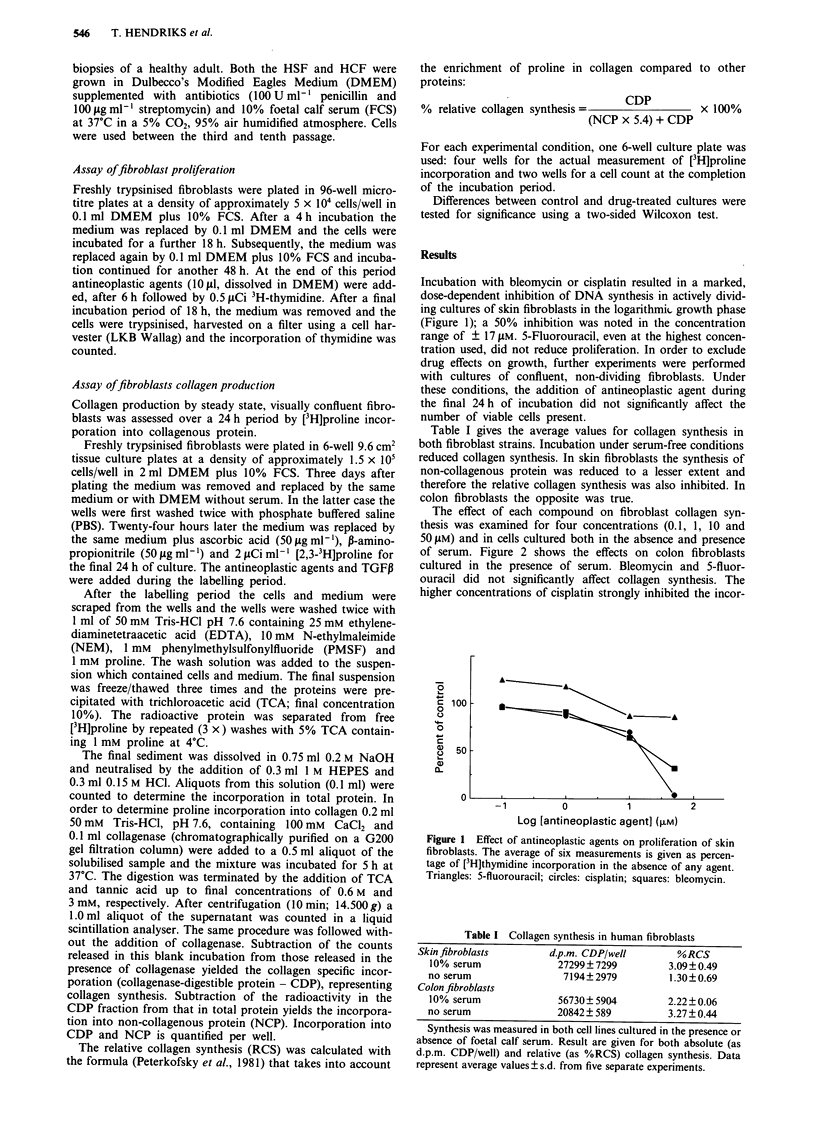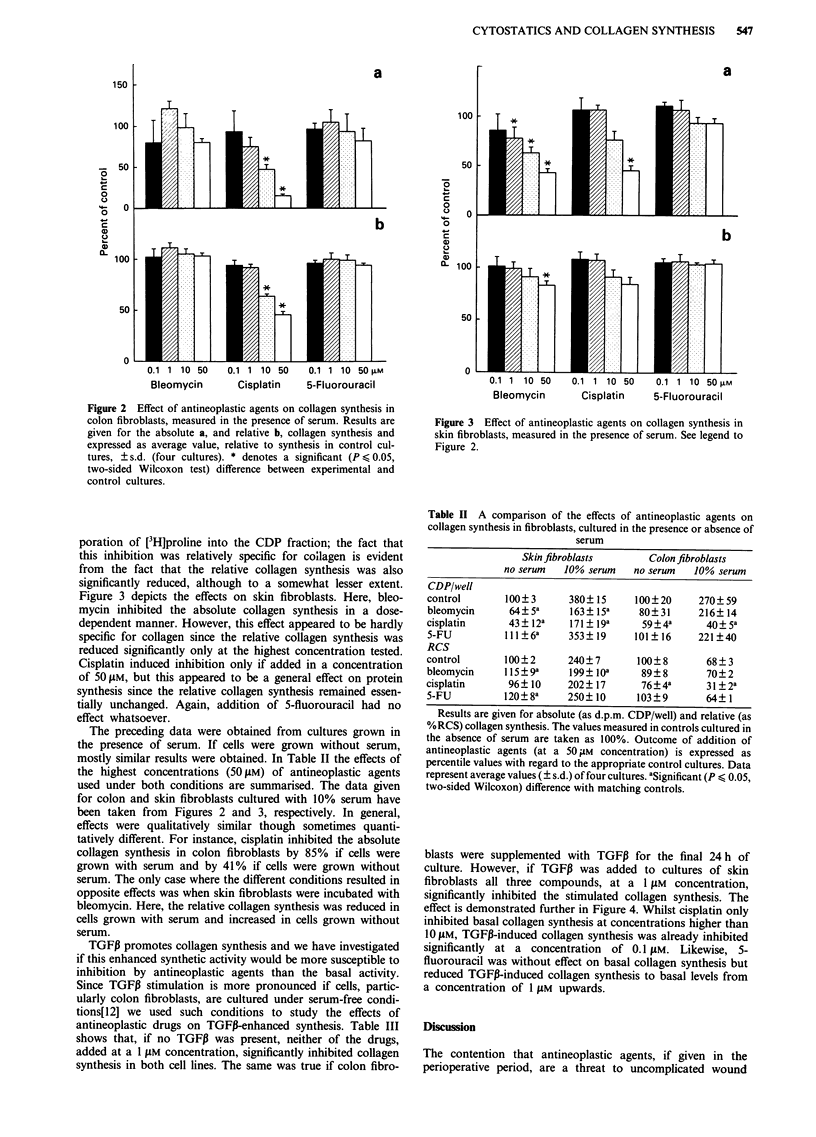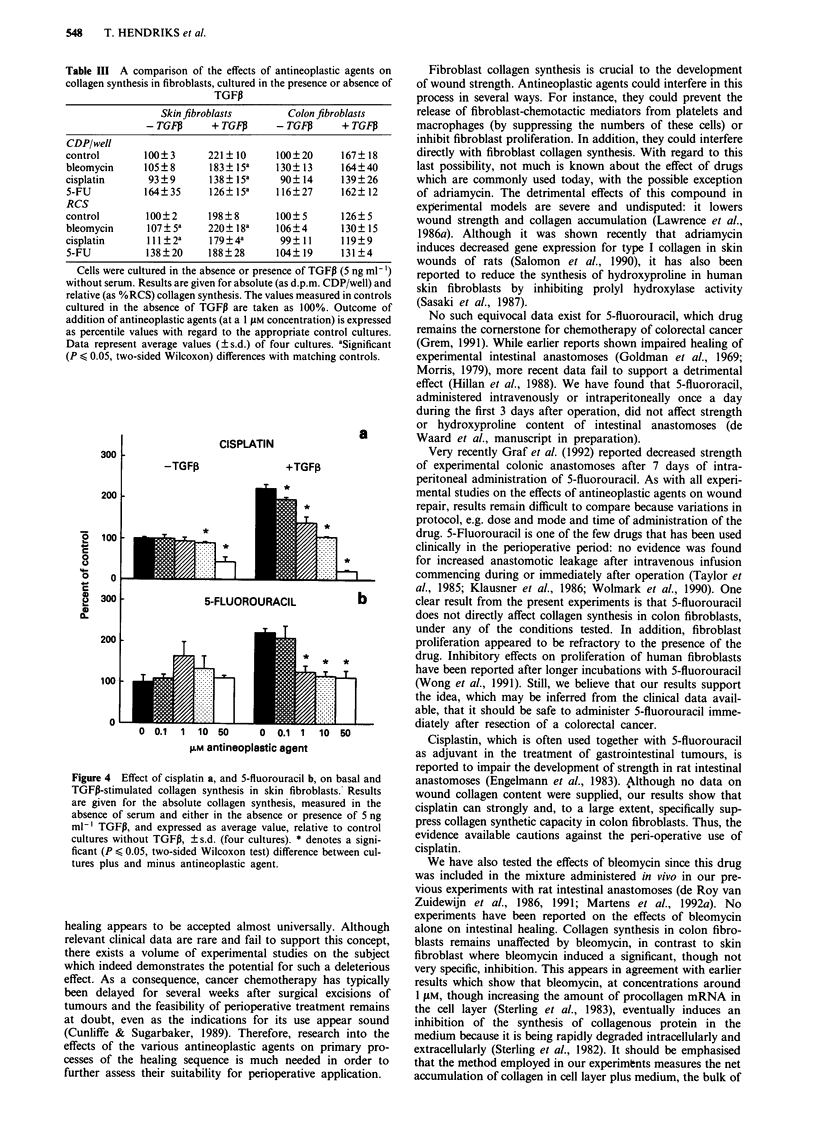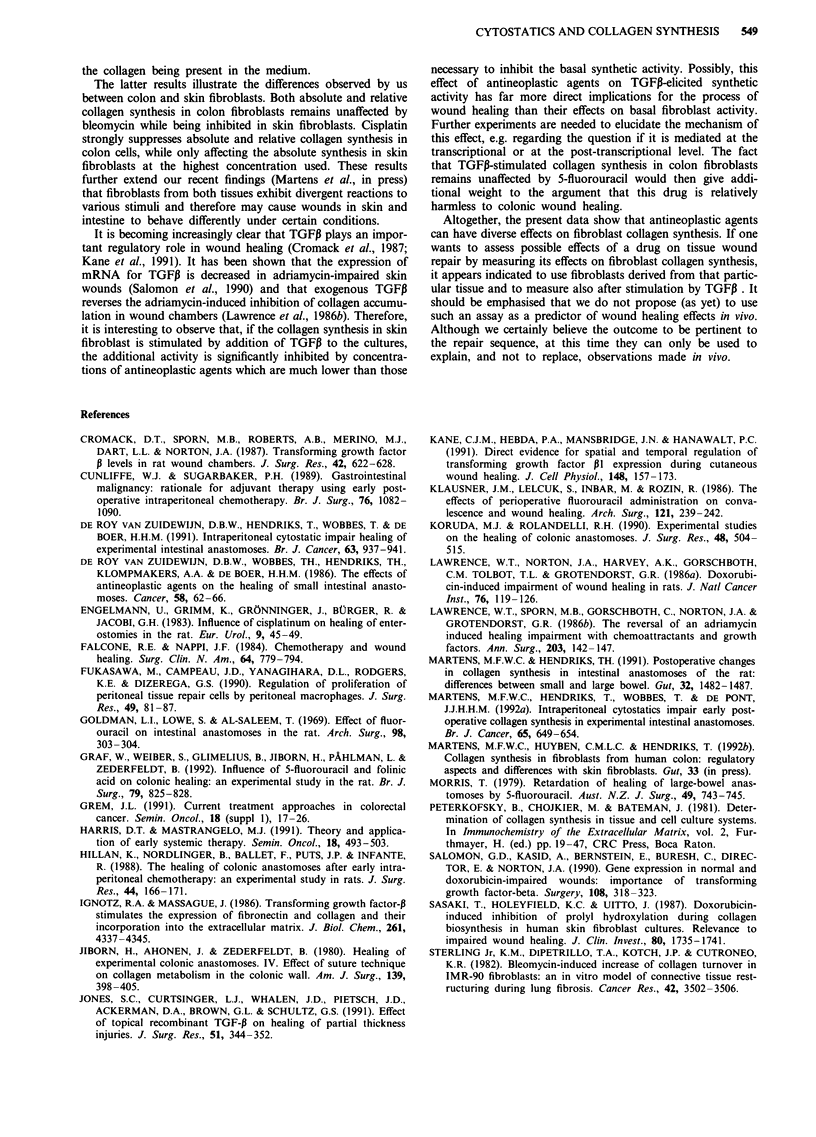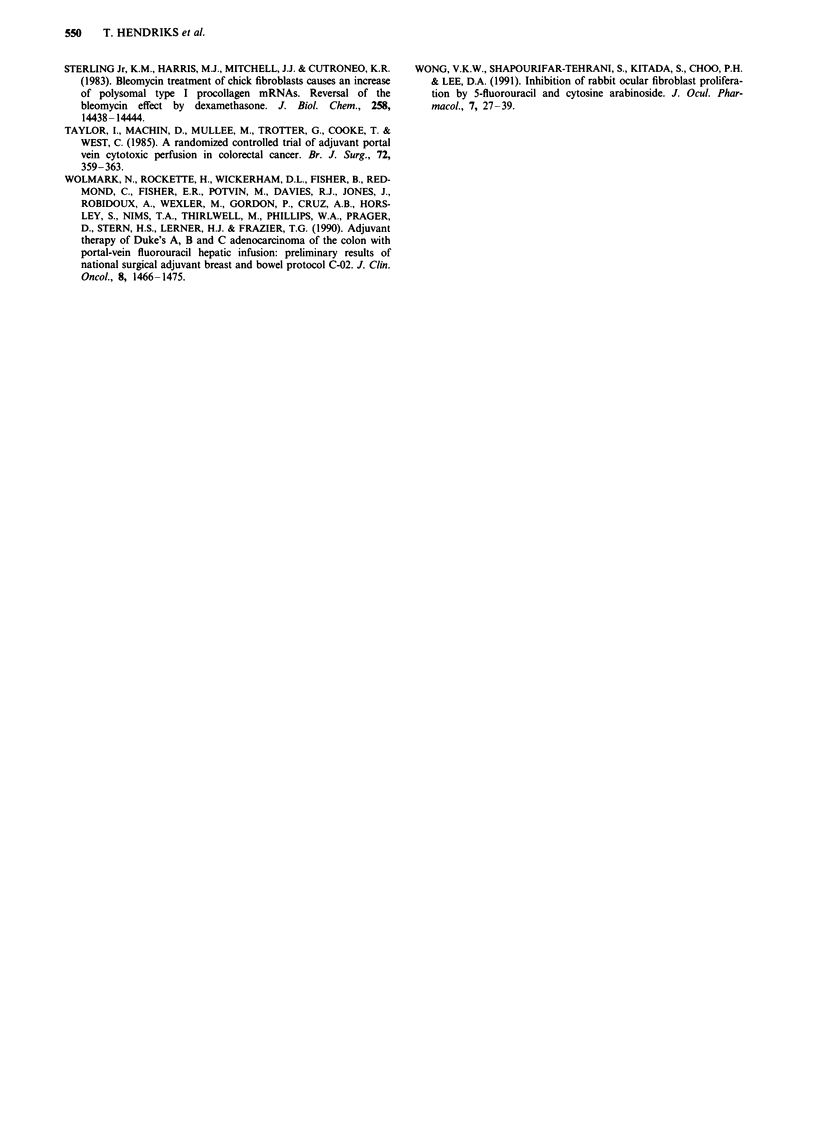# Inhibition of basal and TGF beta-induced fibroblast collagen synthesis by antineoplastic agents. Implications for wound healing.

**DOI:** 10.1038/bjc.1993.100

**Published:** 1993-03

**Authors:** T. Hendricks, M. F. Martens, C. M. Huyben, T. Wobbes

**Affiliations:** Department of General Surgery, University Hospital Nijmegen, The Netherlands.

## Abstract

Antineoplastic drugs, given in the perioperative period, are thought to be a hazard to wound repair. Since fibroblast collagen synthesis is crucial to healing, we examined the effects of bleomycin, cisplatin and 5-fluorouracil on collagen synthesis in confluent cultures of fibroblasts from human colon and skin. The drugs were added in final concentrations between 0.1 and 50 microM. Bleomycin did not affect collagen synthesis in colon fibroblasts but inhibited synthesis in skin fibroblasts. Collagen synthesis in colon fibroblasts was strongly, and specifically, inhibited by cisplatin while synthesis in skin fibroblasts was affected only slightly. 5-Fluorouracil had no effect whatsoever on the collagen synthetic capacity in either colon or skin fibroblasts. If skin fibroblasts were cultured in the presence of transforming growth factor beta (TGF beta), the antineoplastic agents inhibited the TGF beta-stimulated collagen synthesis at far lower concentrations than those needed to suppress non-stimulated synthesis. This effect was not observed in fibroblasts from colon. The possible implications of these observations, as pertain to the use of perioperative chemotherapy, are discussed. Since 5-fluorouracil did not directly affect collagen synthesis in colon fibroblasts under any of the conditions tested it is suggested that the data support the contention that this drug is relatively harmless for intestinal healing.


Br. J. Cancer (1993), 67, 545-550 © Macmillan Press Ltd., 1993

### Inhibition of basal and TGFβ-induced fibroblast collagen synthesis by antineoplastic agents. Implications for wound healing

T. Hendriks, M.F.W.C. Martens, C.M.L.C. Huyben & T. Wobbes

*Department of General Surgery, University Hospital Nijmegen, Nijmegen, The Netherlands.*

**Summary** Antineoplastic drugs, given in the perioperative period, are thought to be a hazard to wound repair. Since fibroblast collagen synthesis is crucial to healing, we examined the effects of bleomycin, cisplatin and 5-fluorouracil on collagen synthesis in confluent cultures of fibroblasts from human colon and skin. The drugs were added in final concentrations between 0.1 and 50 μM.

Bleomycin did not affect collagen synthesis in colon fibroblasts but inhibited synthesis in skin fibroblasts. Collagen synthesis in colon fibroblasts was strongly, and specifically, inhibited by cisplatin while synthesis in skin fibroblasts was affected only slightly. 5-Fluorouracil had no effect whatsoever on the collagen synthetic capacity in either colon or skin fibroblasts. If skin fibroblasts were cultured in the presence of transforming growth factor β (TGFβ), the antineoplastic agents inhibited the TGFβ-stimulated collagen synthesis at far lower concentrations than those needed to suppress non-stimulated synthesis. This effect was not observed in fibroblasts from colon.

The possible implications of these observations, as pertain to the use of perioperative chemotherapy, are discussed. Since 5-fluorouracil did not directly affect collagen synthesis in colon fibroblasts under any of the conditions tested it is suggested that the data support the contention that this drug is relatively harmless for intestinal healing.

At present, surgery remains the only effective treatment modality for patients with malignant gastro-intestinal tumours. However, the occurrence of local and/or regional recurrences constitutes a major problem in the management of such patients. Recurrence rates may be reduced by antineoplastic therapy. Adjuvant treatment for colorectal cancer continues to centre on the use of 5-fluorouracil and regimens that include 5-fluorouracil offer the greatest hope for patients with this malignancy (Grem, 1991). It can be argued that the most suitable time for such therapy would be during or immediately after tumour-reducing surgery (Harris & Mastrangelo, 1991). Since it is generally accepted that most antineoplastic agents used in the perioperative period will impede tissue repair (Falcone & Nappi, 1984), the healing of intestinal anastomoses appears to be at risk after administration of this class of compounds (Koruda & Rolandelli, 1990). Indeed, studies from our laboratory have shown that a combination of bleomycin, cisplatin and 5-fluorouracil, given once a day over 5 consecutive days, severely impairs the development of strength in experimental intestinal anastomoses constructed on the third day of the cytostatic regimen (de Roy van Zuidewijn *et al.*, 1986, 1991). In contrast, administration of 5-fluorouracil alone appears to be less detrimental (Hillan *et al.*, 1988; de Waard *et al.*, manuscript in preparation).

The strength of the intact and the anastomosed bowel wall is derived from collagen fibrils. Normal anastomotic healing is characterized by a strongly enhanced collagen synthetic activity (Jiborn *et al.*, 1980; Martens & Hendriks, 1991). The loss of strength encountered after administration of the cytostatics mixture mentioned above is attended by diminished deposition of collagen in the wound area as a result of a massive inhibition of the collagen synthetic capacity (Martens *et al.*, 1992*a*). Fibroblasts are the major source of newly-formed collagen in a healing wound. Thus, inhibition of wound collagen synthesis by antineoplastic agents may be the result of their effects on fibroblasts chemotaxis and proliferation, either direct or indirect through effects on macrophages, which cells play a pivotal regulatory role in the healing sequence (Fukasawa *et al.*, 1990). This way, the diminished protein synthesis would simply be the result of a reduced presence of fibroblasts in the wound area. In addition, these drugs may directly affect fibroblast protein synthesis. Very few studies are known which report on the specific effects of antineoplastic agents on fibroblast collagen synthesis. We have examined if, and to what extent, bleomycin, cisplatin and 5-fluorouracil inhibit fibroblast collagen synthesis and if these effects are specific for collagen. For this purpose, we have used fibroblasts from both human colon and skin since we found recently that collagen production in these cells may react differently to various stimuli (Martens *et al.*, in press).

We also investigated the effects of the three antineoplastic agents on fibroblast collagen synthesis measured after addition of transforming growth factor β (TGFβ). TGFβ enhances fibroblast collagen production (Ignotz & Massague, 1986) and, if applied topically, promotes wound healing (Jones *et al.*, 1991). Its intrinsic role in the repair process is indicated by its transient and localised expression in healing wounds (Cromack *et al.*, 1987; Kane *et al.*, 1991). Therefore, interference with TGFβ-induced fibroblast collagen synthesis may seriously affect wound repair.

### Materials and methods

### *Materials*

All supplies for cell culture were purchased from Life Technologies (Breda, The Netherlands). The cytostatics used were bleomycin (Lundbeck, Amsterdam, The Netherlands), cisplatin (Lederle, Etten-Leur, The Netherlands) and 5-fluorouracil (Roche, Mijdrecht, The Netherlands). TGFβ1 from bovine bone was a gift from Dr G. Ksander (Celtrix Labs, Palo Alto, USA). L-[2,3-$^3$H]Proline (1,63 TBq/mmol) and [6-$^3$H] thymidine (963 GBq mmol$^{-1}$) were purchased from Amersham International, England. Collagenase (type VII) was obtained from Sigma (St. Louis, USA). All other reagents were of analytical grade (Merck, Darmstadt, Germany).

### *Cell culture*

Normal human colon fibroblasts (HCF) were obtained from the American Type Culture Collection (CRL-1459). Human skin fibroblasts (HSF) were obtained from explants of skin

Correspondence: T. Hendriks, Department of General Surgery, University Hospital Nijmegen, PO Box 9101, 6500 HB Nijmegen, The Netherlands.
Received 27 August 1992; and in revised form 6 October 1992.

biopsies of a healthy adult. Both the HSF and HCF were grown in Dulbecco's Modified Eagles Medium (DMEM) supplemented with antibiotics (100 U $ml^{-1}$ penicillin and 100 μg $ml^{-1}$ streptomycin) and 10% foetal calf serum (FCS) at 37°C in a 5% $CO_2$, 95% air humidified atmosphere. Cells were used between the third and tenth passage.

### *Assay of fibroblast proliferation*

Freshly trypsinised fibroblasts were plated in 96-well microtitre plates at a density of approximately $5 \times 10^4$ cells/well in 0.1 ml DMEM plus 10% FCS. After a 4 h incubation the medium was replaced by 0.1 ml DMEM and the cells were incubated for a further 18 h. Subsequently, the medium was replaced again by 0.1 ml DMEM plus 10% FCS and incubation continued for another 48 h. At the end of this period antineoplastic agents (10 μl, dissolved in DMEM) were added, after 6 h followed by 0.5 μCi $^3$H-thymidine. After a final incubation period of 18 h, the medium was removed and the cells were trypsinised, harvested on a filter using a cell harvester (LKB Wallag) and the incorporation of thymidine was counted.

### *Assay of fibroblasts collagen production*

Collagen production by steady state, visually confluent fibroblasts was assessed over a 24 h period by [$^3$H]proline incorporation into collagenous protein.

Freshly trypsinised fibroblasts were plated in 6-well 9.6 $cm^2$ tissue culture plates at a density of approximately $1.5 \times 10^5$ cells/well in 2 ml DMEM plus 10% FCS. Three days after plating the medium was removed and replaced by the same medium or with DMEM without serum. In the latter case the wells were first washed twice with phosphate buffered saline (PBS). Twenty-four hours later the medium was replaced by the same medium plus ascorbic acid (50 μg $ml^{-1}$), β-aminopropionitrile (50 μg $ml^{-1}$) and 2 μCi $ml^{-1}$ [2,3-$^3$H]proline for the final 24 h of culture. The antineoplastic agents and TGFβ were added during the labelling period.

After the labelling period the cells and medium were scraped from the wells and the wells were washed twice with 1 ml of 50 mM Tris-HCl pH 7.6 containing 25 mM ethylenediaminetetraacetic acid (EDTA), 10 mM N-ethylmaleimide (NEM), 1 mM phenylmethylsulfonylfluoride (PMSF) and 1 mM proline. The wash solution was added to the suspension which contained cells and medium. The final suspension was freeze/thawed three times and the proteins were precipitated with trichloroacetic acid (TCA; final concentration 10%). The radioactive protein was separated from free [$^3$H]proline by repeated (3 ×) washes with 5% TCA containing 1 mM proline at 4°C.

The final sediment was dissolved in 0.75 ml 0.2 M NaOH and neutralised by the addition of 0.3 ml 1 M HEPES and 0.3 ml 0.15 M HCl. Aliquots from this solution (0.1 ml) were counted to determine the incorporation in total protein. In order to determine proline incorporation into collagen 0.2 ml 50 mM Tris-HCl, pH 7.6, containing 100 mM $CaCl_2$ and 0.1 ml collagenase (chromatographically purified on a G200 gel filtration column) were added to a 0.5 ml aliquot of the solubilised sample and the mixture was incubated for 5 h at 37°C. The digestion was terminated by the addition of TCA and tannic acid up to final concentrations of 0.6 M and 3 mM, respectively. After centrifugation (10 min; 14.500 *g*) a 1.0 ml aliquot of the supernatant was counted in a liquid scintillation analyser. The same procedure was followed without the addition of collagenase. Subtraction of the counts released in this blank incubation from those released in the presence of collagenase yielded the collagen specific incorporation (collagenase-digestible protein – CDP), representing collagen synthesis. Subtraction of the radioactivity in the CDP fraction from that in total protein yields the incorporation into non-collagenous protein (NCP). Incorporation into CDP and NCP is quantified per well.

The relative collagen synthesis (RCS) was calculated with the formula (Peterkofsky et al., 1981) that takes into account the enrichment of proline in collagen compared to other proteins:

$$\% \text{ relative collagen synthesis} = \frac{\text{CDP}}{(\text{NCP} \times 5.4) + \text{CDP}} \times 100\%$$

For each experimental condition, one 6-well culture plate was used: four wells for the actual measurement of [$^3$H]proline incorporation and two wells for a cell count at the completion of the incubation period.

Differences between control and drug-treated cultures were tested for significance using a two-sided Wilcoxon test.

### Results

Incubation with bleomycin or cisplatin resulted in a marked, dose-dependent inhibition of DNA synthesis in actively dividing cultures of skin fibroblasts in the logarithmic growth phase (Figure 1); a 50% inhibition was noted in the concentration range of ± 17 μM. 5-Fluorouracil, even at the highest concentration used, did not reduce proliferation. In order to exclude drug effects on growth, further experiments were performed with cultures of confluent, non-dividing fibroblasts. Under these conditions, the addition of antineoplastic agent during the final 24 h of incubation did not significantly affect the number of viable cells present.

Table I gives the average values for collagen synthesis in both fibroblast strains. Incubation under serum-free conditions reduced collagen synthesis. In skin fibroblasts the synthesis of non-collagenous protein was reduced to a lesser extent and therefore the relative collagen synthesis was also inhibited. In colon fibroblasts the opposite was true.

The effect of each compound on fibroblast collagen synthesis was examined for four concentrations (0.1, 1, 10 and 50 μM) and in cells cultured both in the absence and presence of serum. Figure 2 shows the effects on colon fibroblasts cultured in the presence of serum. Bleomycin and 5-fluorouracil did not significantly affect collagen synthesis. The higher concentrations of cisplatin strongly inhibited the incor-

**Figure 1** Effect of antineoplastic agents on proliferation of skin fibroblasts. The average of six measurements is given as percentage of [$^3$H]thymidine incorporation in the absence of any agent. Triangles: 5-fluorouracil; circles: cisplatin; squares: bleomycin.

**Table I** Collagen synthesis in human fibroblasts

| *Skin fibroblasts* | *d.p.m. CDP/well* | *%RCS* |
|---|---|---|
| 10% serum | 27299±7299 | 3.09±0.49 |
| no serum | 7194±2979 | 1.30±0.69 |
| *Colon fibroblasts* | | |
| 10% serum | 56730±5904 | 2.22±0.06 |
| no serum | 20842±589 | 3.27±0.44 |

Synthesis was measured in both cell lines cultured in the presence or absence of foetal calf serum. Result are given for both absolute (as d.p.m. CDP/well) and relative (as %RCS) collagen synthesis. Data represent average values±s.d. from five separate experiments.

**Figure 2** Effect of antineoplastic agents on collagen synthesis in colon fibroblasts, measured in the presence of serum. Results are given for the absolute **a**, and relative **b**, collagen synthesis and expressed as average value, relative to synthesis in control cultures, ±s.d. (four cultures). * denotes a significant ($P \leq 0.05$, two-sided Wilcoxon test) difference between experimental and control cultures.

**Figure 3** Effect of antineoplastic agents on collagen synthesis in skin fibroblasts, measured in the presence of serum. See legend to Figure 2.

poration of [$^3$H]proline into the CDP fraction; the fact that this inhibition was relatively specific for collagen is evident from the fact that the relative collagen synthesis was also significantly reduced, although to a somewhat lesser extent. Figure 3 depicts the effects on skin fibroblasts. Here, bleomycin inhibited the absolute collagen synthesis in a dose-dependent manner. However, this effect appeared to be hardly specific for collagen since the relative collagen synthesis was reduced significantly only at the highest concentration tested. Cisplatin induced inhibition only if added in a concentration of 50 μM, but this appeared to be a general effect on protein synthesis since the relative collagen synthesis remained essentially unchanged. Again, addition of 5-fluorouracil had no effect whatsoever.

The preceding data were obtained from cultures grown in the presence of serum. If cells were grown without serum, mostly similar results were obtained. In Table II the effects of the highest concentrations (50 μM) of antineoplastic agents used under both conditions are summarised. The data given for colon and skin fibroblasts cultured with 10% serum have been taken from Figures 2 and 3, respectively. In general, effects were qualitatively similar though sometimes quantitatively different. For instance, cisplatin inhibited the absolute collagen synthesis in colon fibroblasts by 85% if cells were grown with serum and by 41% if cells were grown without serum. The only case where the different conditions resulted in opposite effects was when skin fibroblasts were incubated with bleomycin. Here, the relative collagen synthesis was reduced in cells grown with serum and increased in cells grown without serum.

TGFβ promotes collagen synthesis and we have investigated if this enhanced synthetic activity would be more susceptible to inhibition by antineoplastic agents than the basal activity. Since TGFβ stimulation is more pronounced if cells, particularly colon fibroblasts, are cultured under serum-free conditions[12] we used such conditions to study the effects of antineoplastic drugs on TGFβ-enhanced synthesis. Table III shows that, if no TGFβ was present, neither of the drugs, added at a 1 μM concentration, significantly inhibited collagen synthesis in both cell lines. The same was true if colon fibro-

**Table II** A comparison of the effects of antineoplastic agents on collagen synthesis in fibroblasts, cultured in the presence or absence of serum

| | *Skin fibroblasts* | | *Colon fibroblasts* | |
|---|---|---|---|---|
| | *no serum* | *10% serum* | *no serum* | *10% serum* |
| *CDP/well* | | | | |
| control | 100±3 | 380±15 | 100±20 | 270±59 |
| bleomycin | 64±5[a] | 163±15[a] | 80±31 | 216±14 |
| cisplatin | 43±12[a] | 171±19[a] | 59±4[a] | 40±5[a] |
| 5-FU | 111±6[a] | 353±19 | 101±16 | 221±40 |
| *RCS* | | | | |
| control | 100±2 | 240±7 | 100±8 | 68±3 |
| bleomycin | 115±9[a] | 199±10[a] | 89±8 | 70±2 |
| cisplatin | 96±10 | 202±17 | 76±4[a] | 31±2[a] |
| 5-FU | 120±8[a] | 250±10 | 103±9 | 64±1 |

Results are given for absolute (as d.p.m. CDP/well) and relative (as %RCS) collagen synthesis. The values measured in controls cultured in the absence of serum are taken as 100%. Outcome of addition of antineoplastic agents (at a 50 μM concentration) is expressed as percentile values with regard to the appropriate control cultures. Data represent average values (±s.d.) of four cultures. [a]Significant ($P \leq 0.05$, two-sided Wilcoxon) difference with matching controls.

blasts were supplemented with TGFβ for the final 24 h of culture. However, if TGFβ was added to cultures of skin fibroblasts all three compounds, at a 1 μM concentration, significantly inhibited the stimulated collagen synthesis. The effect is demonstrated further in Figure 4. Whilst cisplatin only inhibited basal collagen synthesis at concentrations higher than 10 μM, TGFβ-induced collagen synthesis was already inhibited significantly at a concentration of 0.1 μM. Likewise, 5-fluorouracil was without effect on basal collagen synthesis but reduced TGFβ-induced collagen synthesis to basal levels from a concentration of 1 μM upwards.

### Discussion

The contention that antineoplastic agents, if given in the perioperative period, are a threat to uncomplicated wound

**Table III** A comparison of the effects of antineoplastic agents on collagen synthesis in fibroblasts, cultured in the presence or absence of TGFβ

| | *Skin fibroblasts* | | *Colon fibroblasts* | |
|---|---|---|---|---|
| | *− TGFβ* | *+ TGFβ* | *− TGFβ* | *+ TGFβ* |
| *CDP/well* | | | | |
| control | 100±3 | 221±10 | 100±20 | 167±18 |
| bleomycin | 105±8 | 183±15[a] | 130±13 | 164±40 |
| cisplatin | 93±9 | 138±15[a] | 90±14 | 139±26 |
| 5-FU | 164±35 | 126±15[a] | 116±27 | 162±12 |
| *RCS* | | | | |
| control | 100±2 | 198±8 | 100±5 | 126±5 |
| bleomycin | 107±5[a] | 220±18[a] | 106±4 | 130±15 |
| cisplatin | 111±2[a] | 179±4[a] | 99±11 | 119±9 |
| 5-FU | 138±20 | 188±28 | 104±19 | 131±4 |

Cells were cultured in the absence or presence of TGFβ (5 ng ml$^{-1}$) without serum. Results are given for absolute (as d.p.m. CDP/well) and relative (as %RCS) collagen synthesis. The values measured in controls cultured in the absence of TGFβ are taken as 100%. Outcome of addition of antineoplastic agents (at a 1 μM concentration) is expressed as percentile values with regard to the appropriate control cultures. Data represent average values (±s.d.) of four cultures. [a]Significant ($P \leqslant 0.05$, two-sided Wilcoxon) differences with matching controls.

**Figure 4** Effect of cisplatin **a**, and 5-fluorouracil **b**, on basal and TGFβ-stimulated collagen synthesis in skin fibroblasts. Results are given for the absolute collagen synthesis, measured in the absence of serum and either in the absence or presence of 5 ng ml$^{-1}$ TGFβ, and expressed as average value, relative to control cultures without TGFβ, ±s.d. (four cultures). * denotes a significant ($P \leqslant 0.05$, two-sided Wilcoxon test) difference between cultures plus and minus antineoplastic agent.

healing appears to be accepted almost universally. Although relevant clinical data are rare and fail to support this concept, there exists a volume of experimental studies on the subject which indeed demonstrates the potential for such a deleterious effect. As a consequence, cancer chemotherapy has typically been delayed for several weeks after surgical excisions of tumours and the feasibility of perioperative treatment remains at doubt, even as the indications for its use appear sound (Cunliffe & Sugarbaker, 1989). Therefore, research into the effects of the various antineoplastic agents on primary processes of the healing sequence is much needed in order to further assess their suitability for perioperative application.

Fibroblast collagen synthesis is crucial to the development of wound strength. Antineoplastic agents could interfere in this process in several ways. For instance, they could prevent the release of fibroblast-chemotactic mediators from platelets and macrophages (by suppressing the numbers of these cells) or inhibit fibroblast proliferation. In addition, they could interfere directly with fibroblast collagen synthesis. With regard to this last possibility, not much is known about the effect of drugs which are commonly used today, with the possible exception of adriamycin. The detrimental effects of this compound in experimental models are severe and undisputed: it lowers wound strength and collagen accumulation (Lawrence *et al.*, 1986*a*). Although it was shown recently that adriamycin induces decreased gene expression for type I collagen in skin wounds of rats (Salomon *et al.*, 1990), it has also been reported to reduce the synthesis of hydroxyproline in human skin fibroblasts by inhibiting prolyl hydroxylase activity (Sasaki *et al.*, 1987).

No such equivocal data exist for 5-fluorouracil, which drug remains the cornerstone for chemotherapy of colorectal cancer (Grem, 1991). While earlier reports shown impaired healing of experimental intestinal anastomoses (Goldman *et al.*, 1969; Morris, 1979), more recent data fail to support a detrimental effect (Hillan *et al.*, 1988). We have found that 5-fluororacil, administered intravenously or intraperitoneally once a day during the first 3 days after operation, did not affect strength or hydroxyproline content of intestinal anastomoses (de Waard *et al.*, manuscript in preparation).

Very recently Graf *et al.* (1992) reported decreased strength of experimental colonic anastomoses after 7 days of intraperitoneal administration of 5-fluorouracil. As with all experimental studies on the effects of antineoplastic agents on wound repair, results remain difficult to compare because variations in protocol, e.g. dose and mode and time of administration of the drug. 5-Fluorouracil is one of the few drugs that has been used clinically in the perioperative period: no evidence was found for increased anastomotic leakage after intravenous infusion commencing during or immediately after operation (Taylor *et al.*, 1985; Klausner *et al.*, 1986; Wolmark *et al.*, 1990). One clear result from the present experiments is that 5-fluorouracil does not directly affect collagen synthesis in colon fibroblasts, under any of the conditions tested. In addition, fibroblast proliferation appeared to be refractory to the presence of the drug. Inhibitory effects on proliferation of human fibroblasts have been reported after longer incubations with 5-fluorouracil (Wong *et al.*, 1991). Still, we believe that our results support the idea, which may be inferred from the clinical data available, that it should be safe to administer 5-fluorouracil immediately after resection of a colorectal cancer.

Cisplastin, which is often used together with 5-fluorouracil as adjuvant in the treatment of gastrointestinal tumours, is reported to impair the development of strength in rat intestinal anastomoses (Engelmann *et al.*, 1983). Although no data on wound collagen content were supplied, our results show that cisplatin can strongly and, to a large extent, specifically suppress collagen synthetic capacity in colon fibroblasts. Thus, the evidence available cautions against the peri-operative use of cisplatin.

We have also tested the effects of bleomycin since this drug was included in the mixture administered *in vivo* in our previous experiments with rat intestinal anastomoses (de Roy van Zuidewijn *et al.*, 1986, 1991; Martens *et al.*, 1992*a*). No experiments have been reported on the effects of bleomycin alone on intestinal healing. Collagen synthesis in colon fibroblasts remains unaffected by bleomycin, in contrast to skin fibroblast where bleomycin induced a significant, though not very specific, inhibition. This appears in agreement with earlier results which show that bleomycin, at concentrations around 1 μM, though increasing the amount of procollagen mRNA in the cell layer (Sterling *et al.*, 1983), eventually induces an inhibition of the synthesis of collagenous protein in the medium because it is being rapidly degraded intracellularly and extracellularly (Sterling *et al.*, 1982). It should be emphasised that the method employed in our experiments measures the net accumulation of collagen in cell layer plus medium, the bulk of the collagen being present in the medium.

The latter results illustrate the differences observed by us between colon and skin fibroblasts. Both absolute and relative collagen synthesis in colon fibroblasts remains unaffected by bleomycin while being inhibited in skin fibroblasts. Cisplatin strongly suppresses absolute and relative collagen synthesis in colon cells, while only affecting the absolute synthesis in skin fibroblasts at the highest concentration used. These results further extend our recent findings (Martens *et al.*, in press) that fibroblasts from both tissues exhibit divergent reactions to various stimuli and therefore may cause wounds in skin and intestine to behave differently under certain conditions.

It is becoming increasingly clear that TGF$\beta$ plays an important regulatory role in wound healing (Cromack *et al.*, 1987; Kane *et al.*, 1991). It has been shown that the expression of mRNA for TGF$\beta$ is decreased in adriamycin-impaired skin wounds (Salomon *et al.*, 1990) and that exogenous TGF$\beta$ reverses the adriamycin-induced inhibition of collagen accumulation in wound chambers (Lawrence *et al.*, 1986*b*). Therefore, it is interesting to observe that, if the collagen synthesis in skin fibroblast is stimulated by addition of TGF$\beta$ to the cultures, the additional activity is significantly inhibited by concentrations of antineoplastic agents which are much lower than those necessary to inhibit the basal synthetic activity. Possibly, this effect of antineoplastic agents on TGF$\beta$-elicited synthetic activity has far more direct implications for the process of wound healing than their effects on basal fibroblast activity. Further experiments are needed to elucidate the mechanism of this effect, e.g. regarding the question if it is mediated at the transcriptional or at the post-transcriptional level. The fact that TGF$\beta$-stimulated collagen synthesis in colon fibroblasts remains unaffected by 5-fluorouracil would then give additional weight to the argument that this drug is relatively harmless to colonic wound healing.

Altogether, the present data show that antineoplastic agents can have diverse effects on fibroblast collagen synthesis. If one wants to assess possible effects of a drug on tissue wound repair by measuring its effects on fibroblast collagen synthesis, it appears indicated to use fibroblasts derived from that particular tissue and to measure also after stimulation by TGF$\beta$ . It should be emphasised that we do not propose (as yet) to use such an assay as a predictor of wound healing effects *in vivo*. Although we certainly believe the outcome to be pertinent to the repair sequence, at this time they can only be used to explain, and not to replace, observations made *in vivo*.

### References

CROMACK, D.T., SPORN, M.B., ROBERTS, A.B., MERINO, M.J., DART, L.L. & NORTON, J.A. (1987). Transforming growth factor $\beta$ levels in rat wound chambers. *J. Surg. Res.*, **42**, 622–628.

CUNLIFFE, W.J. & SUGARBAKER, P.H. (1989). Gastrointestinal malignancy: rationale for adjuvant therapy using early postoperative intraperitoneal chemotherapy. *Br. J. Surg.*, **76**, 1082–1090.

DE ROY VAN ZUIDEWIJN, D.B.W., HENDRIKS, T., WOBBES, T. & DE BOER, H.H.M. (1991). Intraperitoneal cytostatic impair healing of experimental intestinal anastomoses. *Br. J. Cancer*, **63**, 937–941.

DE ROY VAN ZUIDEWIJN, D.B.W., WOBBES, TH., HENDRIKS, TH., KLOMPMAKERS, A.A. & DE BOER, H.H.M. (1986). The effects of antineoplastic agents on the healing of small intestinal anastomoses. *Cancer*, **58**, 62–66.

ENGELMANN, U., GRIMM, K., GRÖNNINGER, J., BÜRGER, R. & JACOBI, G.H. (1983). Influence of cisplatinum on healing of enterostomies in the rat. *Eur. Urol.*, **9**, 45–49.

FALCONE, R.E. & NAPPI, J.F. (1984). Chemotherapy and wound healing. *Surg. Clin. N. Am.*, **64**, 779–794.

FUKASAWA, M., CAMPEAU, J.D., YANAGIHARA, D.L., RODGERS, K.E. & DIZEREGA, G.S. (1990). Regulation of proliferation of peritoneal tissue repair cells by peritoneal macrophages. *J. Surg. Res.*, **49**, 81–87.

GOLDMAN, L.I., LOWE, S. & AL-SALEEM, T. (1969). Effect of fluorouracil on intestinal anastomoses in the rat. *Arch. Surg.*, **98**, 303–304.

GRAF, W., WEIBER, S., GLIMELIUS, B., JIBORN, H., PÅHLMAN, L. & ZEDERFELDT, B. (1992). Influence of 5-fluorouracil and folinic acid on colonic healing: an experimental study in the rat. *Br. J. Surg.*, **79**, 825–828.

GREM, J.L. (1991). Current treatment approaches in colorectal cancer. *Semin. Oncol.*, **18** (suppl 1), 17–26.

HARRIS, D.T. & MASTRANGELO, M.J. (1991). Theory and application of early systemic therapy. *Semin. Oncol.*, **18**, 493–503.

HILLAN, K., NORDLINGER, B., BALLET, F., PUTS, J.P. & INFANTE, R. (1988). The healing of colonic anastomoses after early intraperitoneal chemotherapy: an experimental study in rats. *J. Surg. Res.*, **44**, 166–171.

IGNOTZ, R.A. & MASSAGUE, J. (1986). Transforming growth factor-$\beta$ stimulates the expression of fibronectin and collagen and their incorporation into the extracellular matrix. *J. Biol. Chem.*, **261**, 4337–4345.

JIBORN, H., AHONEN, J. & ZEDERFELDT, B. (1980). Healing of experimental colonic anastomoses. IV. Effect of suture technique on collagen metabolism in the colonic wall. *Am. J. Surg.*, **139**, 398–405.

JONES, S.C., CURTSINGER, L.J., WHALEN, J.D., PIETSCH, J.D., ACKERMAN, D.A., BROWN, G.L. & SCHULTZ, G.S. (1991). Effect of topical recombinant TGF-$\beta$ on healing of partial thickness injuries. *J. Surg. Res.*, **51**, 344–352.

KANE, C.J.M., HEBDA, P.A., MANSBRIDGE, J.N. & HANAWALT, P.C. (1991). Direct evidence for spatial and temporal regulation of transforming growth factor $\beta$1 expression during cutaneous wound healing. *J. Cell Physiol.*, **148**, 157–173.

KLAUSNER, J.M., LELCUK, S., INBAR, M. & ROZIN, R. (1986). The effects of perioperative fluorouracil administration on convalescence and wound healing. *Arch. Surg.*, **121**, 239–242.

KORUDA, M.J. & ROLANDELLI, R.H. (1990). Experimental studies on the healing of colonic anastomoses. *J. Surg. Res.*, **48**, 504–515.

LAWRENCE, W.T., NORTON, J.A., HARVEY, A.K., GORSCHBOTH, C.M. TOLBOT, T.L. & GROTENDORST, G.R. (1986*a*). Doxorubicin-induced impairment of wound healing in rats. *J. Natl Cancer Inst.*, **76**, 119–126.

LAWRENCE, W.T., SPORN, M.B., GORSCHBOTH, C., NORTON, J.A. & GROTENDORST, G.R. (1986*b*). The reversal of an adriamycin induced healing impairment with chemoattractants and growth factors. *Ann. Surg.*, **203**, 142–147.

MARTENS, M.F.W.C. & HENDRIKS, TH. (1991). Postoperative changes in collagen synthesis in intestinal anastomoses of the rat: differences between small and large bowel. *Gut*, **32**, 1482–1487.

MARTENS, M.F.W.C., HENDRIKS, T., WOBBES, T. & DE PONT, J.J.H.H.M. (1992*a*). Intraperitoneal cytostatics impair early postoperative collagen synthesis in experimental intestinal anastomoses. *Br. J. Cancer*, **65**, 649–654.

MARTENS, M.F.W.C., HUYBEN, C.M.L.C. & HENDRIKS, T. (1992*b*). Collagen synthesis in fibroblasts from human colon: regulatory aspects and differences with skin fibroblasts. *Gut*, **33** (in press).

MORRIS, T. (1979). Retardation of healing of large-bowel anastomoses by 5-fluorouracil. *Aust. N.Z. J. Surg.*, **49**, 743–745.

PETERKOFSKY, B., CHOJKIER, M. & BATEMAN, J. (1981). Determination of collagen synthesis in tissue and cell culture systems. In *Immunochemistry of the Extracellular Matrix*, vol. 2, Furthmayer, H. (ed.) pp. 19–47, CRC Press, Boca Raton.

SALOMON, G.D., KASID, A., BERNSTEIN, E., BURESH, C., DIRECTOR, E. & NORTON, J.A. (1990). Gene expression in normal and doxorubicin-impaired wounds: importance of transforming growth factor-beta. *Surgery*, **108**, 318–323.

SASAKI, T., HOLEYFIELD, K.C. & UITTO, J. (1987). Doxorubicin-induced inhibition of prolyl hydroxylation during collagen biosynthesis in human skin fibroblast cultures. Relevance to impaired wound healing. *J. Clin. Invest.*, **80**, 1735–1741.

STERLING Jr, K.M., DIPETRILLO, T.A., KOTCH, J.P. & CUTRONEO, K.R. (1982). Bleomycin-induced increase of collagen turnover in IMR-90 fibroblasts: an in vitro model of connective tissue restructuring during lung fibrosis. *Cancer Res.*, **42**, 3502–3506.

STERLING Jr, K.M., HARRIS, M.J., MITCHELL, J.J. & CUTRONEO, K.R. (1983). Bleomycin treatment of chick fibroblasts causes an increase of polysomal type I procollagen mRNAs. Reversal of the bleomycin effect by dexamethasone. *J. Biol. Chem.*, **258**, 14438–14444.

TAYLOR, I., MACHIN, D., MULLEE, M., TROTTER, G., COOKE, T. & WEST, C. (1985). A randomized controlled trial of adjuvant portal vein cytotoxic perfusion in colorectal cancer. *Br. J. Surg.*, **72**, 359–363.

WOLMARK, N., ROCKETTE, H., WICKERHAM, D.L., FISHER, B., REDMOND, C., FISHER, E.R., POTVIN, M., DAVIES, R.J., JONES, J., ROBIDOUX, A., WEXLER, M., GORDON, P., CRUZ, A.B., HORSLEY, S., NIMS, T.A., THIRLWELL, M., PHILLIPS, W.A., PRAGER, D., STERN, H.S., LERNER, H.J. & FRAZIER, T.G. (1990). Adjuvant therapy of Duke's A, B and C adenocarcinoma of the colon with portal-vein fluorouracil hepatic infusion: preliminary results of national surgical adjuvant breast and bowel protocol C-02. *J. Clin. Oncol.*, **8**, 1466–1475.

WONG, V.K.W., SHAPOURIFAR-TEHRANI, S., KITADA, S., CHOO, P.H. & LEE, D.A. (1991). Inhibition of rabbit ocular fibroblast proliferation by 5-fluorouracil and cytosine arabinoside. *J. Ocul. Pharmacol.*, **7**, 27–39.